# Baicalein Potentiated M1 Macrophage Polarization in Cancer Through Targeting PI3Kγ/ NF-κB Signaling

**DOI:** 10.3389/fphar.2021.743837

**Published:** 2021-08-25

**Authors:** Shan He, Shangshang Wang, Suqing Liu, Zheng Li, Xiao Liu, Jinfeng Wu

**Affiliations:** Department of Dermatology, Huashan Hospital, Fudan University, Shanghai, China

**Keywords:** baicalein, tumor-associated macrophages, polarization, PI3Kγ, NF-k B

## Abstract

Baicalein is one of the bioactive compounds extracted from *Scutellaria baicalensis*. Recent studies indicated the antitumor effects of baicalein, however, the underlying mechanisms are needed to be further determined. In this study, we found that baicalein could inhibit the tumor growth in mice models of breast cancer and melanoma and worked as an immunomodulator to promote the infiltration of tumor-associated macrophages (TAMs) and skew the TAMs towards the M1-like phenotype. Baicalein also induced M1-like phenotype polarization in THP-1-derived macrophages. Meanwhile, the expression of pro-inflammatory factors associated with M1 macrophages, including TNF-α, IL-1β, CXCL9 and CXCL10, were increased after baicalein treatment. Mechanistically, the RNA-seq data suggested that baicalein potentiated the M1 macrophage polarization *via* the NF-κB/TNF-α signaling pathway. ELISA and confocal microscopy assay confirmed that baicalein significantly induced the production of TNF-α and the activation of NF-κB, while TNF-α neutralization inhibited baicalein-induced macrophage polarization toward M1, and NF-κB P65 knock-down suppressed baicalein-induced TNF-α production in THP-1-derived macrophages. Phosphoinositide 3-kinase (PI3k) γ has been reported as a key molecule in macrophage polarization, and inhibition of PI3Kγ activates the NF-κB-related inflammatory signals. Our pharmacological network analysis predicted that PI3Kγ might be one of the major targets of baicalein. The results from the docking program and surface plasmon resonance (SPR) confirmed that baicalein displayed good binding activity to PI3Kγ. We further found that baicalein not only exhibited a direct inhibitory effect on the protein kinase activity of PI3Kγ, but also reduced the mRNA and protein expression of PI3Kγ, indicating that baicalein might be a novel PI3Kγ inhibitor. In summary, baicalein mediated the TAMs skewing to M1-TAMs, and then retarded tumor growth. These effects, at least in part, were linked to the PI3Kγ/NF-κB signaling.

## Introduction

Cancer, one of the leading causes of death globally, has always been a serious threat to public health and a formidable challenge for the current health care system. Solid tumors, such as breast cancer and melanoma, can be treated with conventional therapeutic options including surgical resection, radiotherapy, chemotherapy, as well as increasing numbers of treatment strategies developed in the past decades. However, due to the uncontrolled tumor progression, high rate of tumor metastasis, and drug resistance, a fraction of patients fail to benefit from the current treatment modalities.

TAMs represent one of the major cellular components in the Tumor Microenvironment (TME) and play a critical role in tumor pathogenesis (Ruffell and Coussens, 2015). In response to specific signals within the TME, macrophages can be activated and categorized as two main phenotypes: M1 macrophages (classically activated macrophages) and M2 macrophages (alternatively activated macrophages). The M1 phenotype typically produces tumor necrosis factor (TNF), interleukin (IL)-12 and interferon-γ (IFN-γ) inducible chemokines C-X-C motif chemokine ligand (CXCL) 9 and CXCL10 to exert pro-inflammatory and antitumor effects, whereas M2 macrophages, mainly identified by the production of IL-10 and Arg-1, are known to inhibit inflammatory responses and promote tumor progression (Mantovani et al., 2002; Murray et al., 2014; Lee et al., 2019). Within the TME, tumor cells facilitate the macrophage polarization towards M2-TAM. The decrease in M1-TAM and M1-associated cytokines and chemokines such as TNF-α and CXCL10 have also been demonstrated (Mantovani et al., 2002; De Henau et al., 2016; Kaneda et al., 2016). Thus, reeducating the TAMs to the M1 phenotype might be a promising strategy for cancer treatment (Li et al., 2019).

Traditional Chinese medicine (TCM) has been developed and applied to treating diseases for over 2,500 years (Wang et al., 2020). *Scutellaria baicalensis* (also known as Chinese Huang Qin) is one of the most widely used herbs in TCM formula for treating various diseases, such as diarrhea, insomnia, and acute respiratory infection. Baicalein, one of the major bioactive compounds isolated from *Scutellaria baicalensis,* has been demonstrated multiple pharmacological effects, including anti-inflammatory, antiviral, anti-adipogenesis and cardiovascular protective effects (Liu et al., 2016; Nik Salleh et al., 2020). Recent studies revealed that baicalein exerted anti-tumor activities in various solid tumors, such as non-small-cell lung cancer (NSCLC), and breast cancer (Yan et al., 2018; Zhao et al., 2018; Nik Salleh et al., 2020; Zhang et al., 2020). Previous reports have shown the underlying mechanisms of baicalein on suppressing the proliferation and invasion of cancer cells by inducing autophagy and apoptosis, targeting arachidonic acid pathway, inhibiting AMPK or PI3K/AKT signaling pathways (Yarla et al., 2016; Yan et al., 2018; Ke et al., 2019; Deng et al., 2020).

However, considering that the TAMs infiltrating in the TME play a key role in tumor growth and metastasis as well as prognosis after treatments (Martinez and Gordon, 2014), we evaluated the effects of baicalein on tumor immunity, especially TAMs in the TME. In addition, recent studies demonstrated PI3Kγ as an important molecule in switching the immunosuppressive and immunostimulatory functions of TAMs, which was partly associated with the NF-κB pathway (Li et al., 2019). In this study, we also clarified the underlying mechanism of baicalein-induced TAM polarization through the PI3Kγ/NF-κB pathway.

## Material and Methods

### Reagents

**FIGURE 1:**
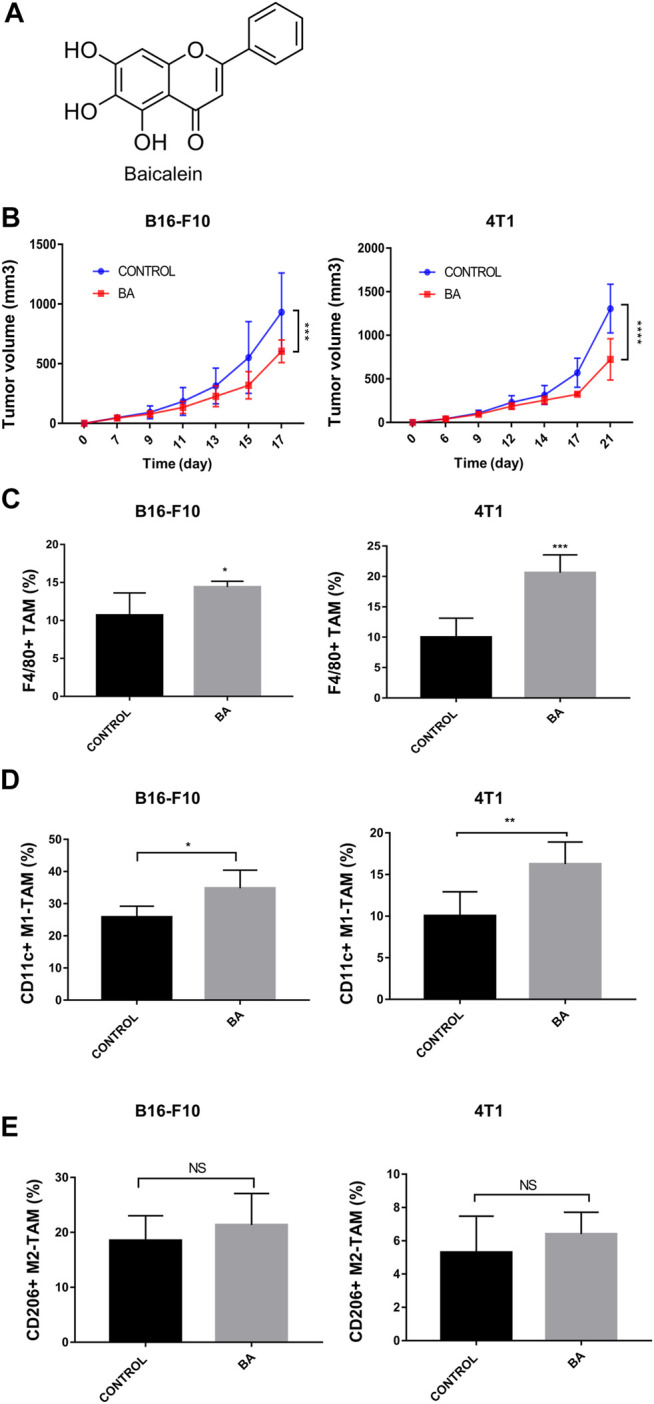
Baicalein inhibited tumor growth and promoted M1 macrophage polarization, **(A)** chemical structure of baicalein. **(B)** tumor growth curves in mice bearing B16-F10 melanoma and 4T1 breast cancer. Baicalein (50 mg/kg) was treated i.p. every other day. The proportion of tumor-infiltrating macrophages and the subtypes of TAMs in mice tumor tissues after treatment were quantified by flow cytometry. Corresponding quantification results of F4/80 + TAMs **(C)**, CD11c + M1-TAMs **(D)** and CD206 + M2-TAMs **(E)** were shown. Data are presented as mean ± SD and were obtained from at least independent experiments. *p < 0.05, **p < 0.01, ***p < 0.001 for comparison with control group. NS, no significance. BA = baicalein.

Baicalein (BA, C_15_H_10_O_5_, molecular weight: 270.24, purity ≥99%, Figure 1A) was purchased from Shanghai Ronghe Co. (Shanghai, China), and was dissolved in dimethyl sulfoxide (DMSO) and kept in −80°C. The following antibodies were obtained from Biolegend (San Diego, CA, United States): anti-mouse CD206-AF647 (Cat#141712), anti-mouse Foxp3-PE (Cat#320008), anti-mouse CD4-PerCP/Cy5.5 (Cat#100434), anti-mouse CD11b-FITC (Cat#101206), anti-mouse CD25-APC (Cat#101910), anti-mouse CD45-APC/Cy7 (Cat#157617), anti-mouse CD11c-PE/Cy7 (Cat#117318), anti-mouse F4/80-PE (Cat#123110), anti-mouse CD8a-FITC (Cat#155004), and anti-mouse CD3ε-PE/Cy7 (Cat#155706). Fixable Viability Stain (FVS) 700 was purchased from BD Bioscience (San Jose, CA, United States, Cat#564997). IFN-γ was supplied by Peprotech (Cranbury, NJ, United States, Cat#500-M90). Phorbol 12-myristate 13-acetate (PMA) was purchased from Lianke (Hangzhou, China, Cat#70-CS0001). Lipopolysaccharide (LPS) was obtained from Sigma-Aldrich (Merck Life Science, Darmstadt, Germany, SKU#L3129-25 MG).

### Cell Lines and Macrophage Polarization

The B16-F10 murine melanoma cell line, 4T1 murine breast cancer cell line, and the human monocyte cell line THP-1 were purchased from the Cell Bank of the China Science Academy (Shanghai, China). 4T1 cells and THP-1 cells were cultured in the RPMI-1640 medium (Kaiji, Nanjing, China), and B16-F10 cells were maintained in the DMEM medium. All of these mediums were supplemented with 10% fetal bovine serum (FBS, ScienCell, Carsbad, CA, United States, Cat#0500). The differentiation of THP-1 into M0 macrophages was induced by incubating with 320 nmol/L PMA for 24 h, and then the cells were treated with 100 nmol/L PMA plus 20 ng/ml IFN-γ and 100 ng/ml LPS for 48 h to obtain M1 macrophages.

### Flow Cytometry Assay

For analysis of tumor-infiltrating immune cells, tumors were excised, cut into small pieces, filtered through 300 mesh screens, and washed twice to obtain single-cell suspensions, and then the cells were treated with 1X red blood cell (RBC) lysis buffer. FVS700 was used for the discrimination of viable from non-viable cells. Mononuclear cells were then stained with anti-mouse antibodies against CD45, F4/80, CD11b, CD11c and CD206, and T cells were labeled with antibodies against CD45, CD3, CD4, CD8, CD25, Foxp3, according to the manufacture protocols. For analysis of THP-1 cells-derived M1 monocytes, antibody against CD86 was used (BD Bioscience, San Jose, CA, United States, Cat#560957). The cells were sorted on a BD LSRFortessa and analyzed using the FlowJo software (Ashland, OR, United States).

### Quantitative RT-PCR Assay

Total RNA was extracted by Trizol (Invitrogen; Thermo Fisher Scientific, Inc., Waltham, MA, United States, Cat#15596018) and was reversed to cDNA using the Prime Script RT reagent Kit (TaKaRa, Japan, Cat# RR036B). The quantitative real-time PCR (qRT-PCR) was conducted according to the instructions of TB Premix Ex TaqⅡRT-PCT Kit (TaKaRa, Japan, Cat#RR820B) and data was analyzed by the 2^−ΔΔCt^ method relative to the expression of actin. Sequences of the primers in this study were listed in Supplementary Table S1.

### RNA-Seq Assay

Total RNA of cultured cells was isolated by Trizol. The concentration and purity of RNA were detected by a Bioanalyzer 4,200 (Agilent, Santa Clara, CA, United States). Analysis of RNA-seq was performed as described in our previous article (Li et al., 2020).

### SiRNA Transfection

P65 siRNA was produced by Genepharma, Co. (Shanghai, China). The following sequences of siRNA were used for P65 gene knockdown: 5′-CGG AUU GAG GAG AAA CGU ATT-3′(sense) and 5′-UAC GUU UCU CCU CAA UCC GTT-3′(anti-sense). Nonspecific control oligo was used as negative controls. P65 siRNA was transfected into the cells at a final concentration of 10 nM using Lipofectamine 2,000 reagent (Invitrogen, Cat#11668030) according to the manufacturer’s instructions. P65 knockdown was tested by qRT-PCR 24 h after the transfection.

### Fluorescence Confocal Microscopy Assay

Cells were seeded on coverslips in 24 wells plates. According to the instruction of the Cellular NF-κB Translocation Kit (Beyotime Biotech, Jiangsu, China, Cat#SN368), cells were fixed and then incubated with blocking buffer for 1 h. After washing, cells were incubated with NF-κB p65 antibody for 1 h at room temperature, then a Cy3 fluorescence-labeled secondary antibody was used. After that, coverslips were then incubated with phosphoinositide 3-kinase (DAPI) for 5 min. Finally, the coverslips were examined with a confocal laser scanning microscopy (Zeiss, Thornwood, NY).

### Western Blotting

After treatment, the cells were resuspended in RIPA lysis buffer (Beyotime). The cellular proteins were electroblotted onto a PVDF membrane following separation *via* 10% SDS-polyacrylamide gel electrophoresis. The immunoblots were incubated with antibodies against P65 (molecular weight (MW): 65 kDa), p-P65 (MW: 65–80 kDa), IκB (MW: 36 kDa), *p* (MW: 35 kDa), and actin (MW: 42 kDa) antibodies (all from Abcam, Cambridge, United Kingdom, Cat#ab32536, Cat#ab183559, Cat#ab32518, Cat#ab133462, Cat#ab179467, respectively). Antibody against PI3Kγ (MW: 110 kDa) was purchased from Cell Signaling Technologies (MA, United States, Cat#4252S). Images were acquired by Tanon 5200-multi chemiluminescent imaging system (Tanon Science and Technology Co. Ltd., Shanghai, China).

### ELISA

The supernatant of tumor tissues or cells was collected, and TNF-α levels in the supernatant were measured using enzyme-linked immunosorbent assay (ELISA) kits (Invitrogen, Cat#BMS223-4, Cat#88–7324-77) according to the manufactures’ instructions. To analyze the levels of TNF-α, 96-well Coat corning™ Costar™ 9018 ELISA plates were coated with diluted capture antibody overnight at 4°C. After washing in washing buffer, the wells were blocked with ELISA/ELISPOT diluent at room temperature for 1 h. Next, standards and diluted samples were added to appropriate wells, and incubated at room temperature for 2 h. After three times of washing, the wells were incubated with the detection antibody for 1 h. Diluted Streptavidin-HRP and TMB solutions were added and incubated accordingly following the instruction. After adding the stop solution, the signals were read at 450 nm.

### Molecular Docking Analysis

GOLD Suite v5.3 was used to predict the binding site of baicalein (CID_5281605) on PI3Kγ (PDB ID:2a4z). Analysis of the binding ability and ligand binding energy with target protein was performed as described in our previous article (Wu et al., 2016).

### SPR Assay

The interaction between baicalein and PI3Kγ (Abcam, Cat#ab268859) was evaluated by SPR (Nicoya Lifescience, Canada). Compounds were diluted with activation buffer. The NTA sensor chip was installed on the OpenSPR instrument following the standard procedure, and the sensor chip was loaded with NiCl_2,_ followed by injection with His-tagged PI3Kγ protein. Different concentrations of baicalein were added to pass over the NTA sensor chip at a flow rate of 20 μl/min in the running buffer (1 mM PBS). Trace Drawer software (Ridgeview Instruments AB, The Kingdom of Sweden) was used for calculating and analyzing the kinetic parameters of the binding reaction.

### PI3Kγ Kinase Assay

For the measurement of the kinase activity of PI3Kγ, ADP-Glo kinase assay kit (Promega, Madison, WI, United States, Cat#V910) and PI3Kγ (p110γ) assay kit (BPS Bioscience, San Diego, CA, Cat#79803) were used. PI3Kγ inhibitor AS-605240 (Selleck Chemicals, Houston, TX, United States, Cat#S1410) was used as a positive control. The concentration of baicalein was chosen by eight serial dilutions starting at 200 μM and subsequently diluted 4-fold in DMSO. After adding diluted compounds and ATP/substrate mixture to 96-well assay plates, PI3Kγ kinase was added to start the reaction. The plates were incubated for 40 min at 30°C and then treated with ADP-Glo reagent. After incubation for another 40 min at room temperature, kinase detection reagent was added. Luminescence was used for detecting the production of ADP to further calculate the percentage of inhibition.

### *In vivo* Studies

Six-week-old female C57BL/6 J and Balb/c mice were purchased from Zhejiang Vital River Laboratory Animal Technology Co. (Zhejiang, China). For the B16-F10 melanoma tumor model, 2.0 x 10^5^ B16-F10 cells were injected intradermally in the right flank of C57BL/6 J mice. For the 4T1 breast tumor model, 5 x 10^5^ 4T1 cells were injected subcutaneously in the right flank of Balb/c mice. In both models, baicalein (50 mg/kg) was injected intraperitoneally (i.p.) every other day. The treatment was initiated on day 7 and ended on day 21. Tumor size was measured every second or third day with a digital caliper and was calculated as ab^2^/2 (a is the longest diameter and b is the shortest diameter). On day 22, animals were sacrificed and the tumors were removed.

### Statistical Methods

All the results of quantitative assays were shown as mean ± SD from three independent experiments. Statistical analysis was performed using student’s t-test for comparisons between two groups, one-way ANOVA, and subsequent Tukey’s post-hoc analysis for comparison of more than two independent groups by GraphPad Prism 7 software. *p* < 0.05 was considered to indicate a statistically significant difference.

## Results

### Baicalein Inhibited Tumor Growth by Promoting M1 Macrophage Polarization

To evaluate the anti-tumor effects of baicalein *in vivo*, we utilized mice with the 4T1 breast tumor and B16-F10 melanoma. As shown in Figure 1B, baicalein successfully inhibited the tumor growth in both 4T1 and B16-F10 tumor-bearing mice (Figure 1B, ***p = 0.0008, ****p < 0.0001, respectively). Next, we performed flow cytometry analysis to explore the influence of baicalein on the immune cells infiltrating in the tumors. As demonstrated in Figures 1C,D, baicalein treatment promoted the infiltration of F4/80-positive TAM, and the proportion of infiltrated M1 macrophages in TAM was also increased after the baicalein treatment (Figure 1C, *p = 0.0496, ***p = 0.0005; Figure 1D, *p = 0.0344, **p = 0.0075). However, baicalein did not alter the percentage of M2 macrophages in TAMs (Figure 1E, NS: *p* = 0.4649, *p* = 0.3618). Since the role of T cells cannot be ignored in tumor growth and progression, we also analyzed CD4^+^ T cells, CD8^+^ cytotoxic T cells and CD4^+^CD25^+^Foxp3^+^ Treg cells in tumor-bearing mice. It is worth noting that baicalein did not influence the percentages of these subpopulations of T cells (Supplementary Figure S2).

### Baicalein Potentiated M1 Macrophage Polarization *in vitro*

**FIGURE 2:**
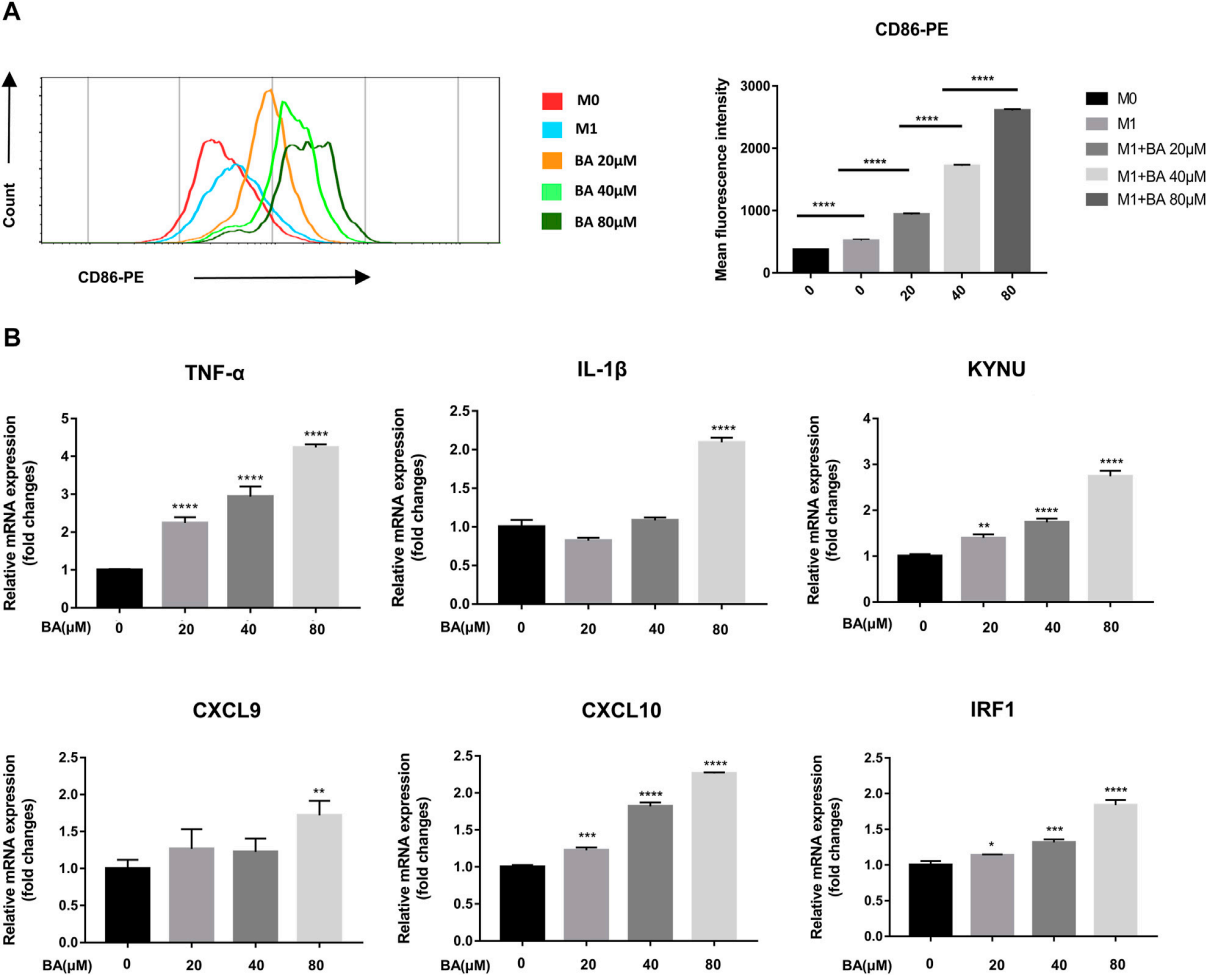
Baicalein promoted M1 macrophage polarization *in vitro*. THP-1-derived macrophages were treated with LPS and IFN-γ with or without different concentrations of baicalein for 24 h or 48 h. **(A)** the expression of CD86, the M1 macrophage marker, was quantified by flow cytometry. FlowJo software V10.0 was used for analyzing the mean fluorescence intensity data. **(B)** qRT-PCR was used to examine the mRNA expression levels of M1-associated genes including TNF-α, IL-1β, KYNU, CXCL9, CXCL10, and IRF1. Data are presented as mean ± SD and are obtained from at least independent experiments. *p < 0.05, **p < 0.01, ***p < 0.001, ****p < 0.0001, for comparison with control group. NS, no significance. BA = baicalein.

Although baicalein modulated macrophage polarization towards the M1 phenotype *in vivo*, whether baicalein could directly act on macrophage polarization remains unknown. Next, we examined the direct effects of baicalein on the polarization and function of macrophages *in vitro*. THP-1-generated macrophages have been recognized as a reasonable model of macrophage polarization (Heideveld et al., 2020), and human M1 macrophages can be identified by surface marker CD86 (Mantovani et al., 2002). Therefore, we examined the expression of CD86 in THP-1 derive M1 macrophages by flow cytometry and found that the expression of CD86 was enhanced by baicalein in a dose-dependent manner (Figure 2A, ****p < 0.0001). In addition, the mRNA expressions of M1-related pro-inflammatory cytokines and other factors (Murray et al., 2014), including TNF-α, IL-1β, CXCL9, CXCL10, KYNU and IRF-1, were significantly up-regulated after baicalein treatment (Figure 2B, *p < 0.05, **p < 0.01, ****p < 0.0001).

### Baicalein Promoted Macrophage Skewing to M1 Phenotype *via* NF-κB Signaling Pathway

**FIGURE 3:**
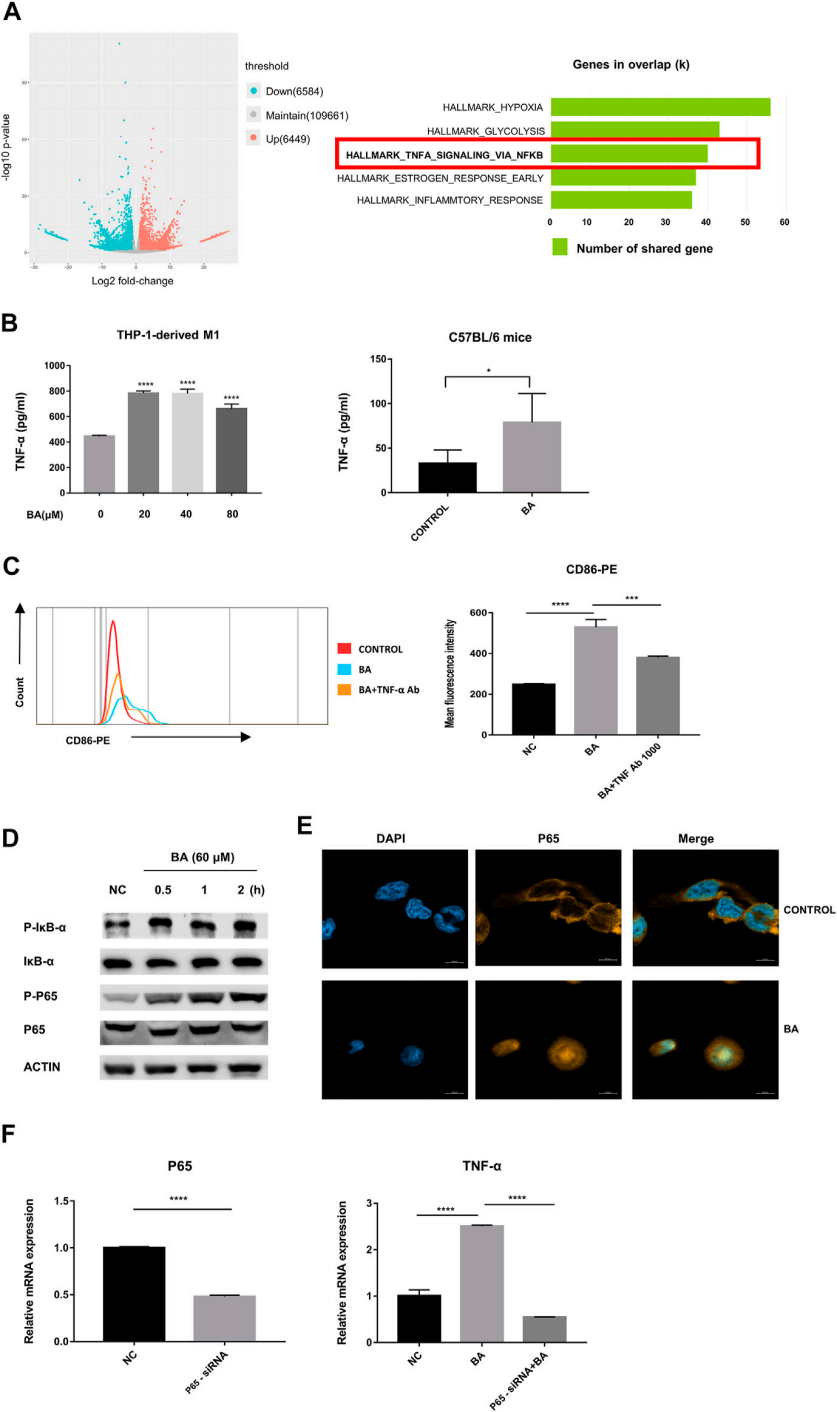
Baicalein promoted M1 macrophage polarization via NF-κB/TNF-α signaling pathways. **(A)** left, RNA-seq analysis was performed and the volcano plot was demonstrated. **(A)** right, results of Hallmark analysis on up-regulated genes in the baicalein group compared with that in the control group. **(B)** the levels of TNF-α in the supernatant of THP-1-derived M1 macrophage (left) and tumor tissues (right) were measured by ELISA. **(C)** TNF-α neutralizing antibody (1,000 ng/ml) was used to block TNF-α and the percentage of CD86-positive macrophages was detected by flow cytometry. **(D)** M1 macrophages derived from THP-1 were treated with baicalein (60 μM) for varying times as indicated. The cytoplasmic levels of *p*-IκB, IκB, P65, and p-P65 were measured by western blotting. After exposure to BA for 30 min, the expression levels of p-p65 and *p*-IκB increased, with higher levels being sustained until 120 min. **(E)** nuclear translocation of NF-κB P65 in THP-1-derived M1 macrophages was detected by fluorescent staining and confocal laser scanning microscopy. Cells were treated with baicalein (60 μM) for 4 h. The expression of P65 was labeled with a primary specific antibody and a subsequent Cy3-conjugated secondary antibody (yellow). The nucleus was stained by DAPI (blue). **(F)** P65 siRNA was used to knock down P65 in M1 macrophages. QRT-PCR was used to detect the efficiency of P65 knockdown (left) and the mRNA expression of TNF-α in transfected cells (right). All experiments were repeated three times independently. Data are presented as mean ± SD. *p < 0.05, ***p < 0.001, ****p < 0.0001, for comparison with control group. NS, no significance. BA = baicalein, NC = negative control.

To determine the mechanism of baicalein-induced M1 polarization, we performed RNA-seq analysis and found that 6,584 genes were significantly down-regulated and 6,449 were markedly up-regulated in volcano plots. Moreover, the Hallmark analysis indicated that TNF-α *via* NF-κB signaling pathway was notably inhibited after baicalein treatment (Figure 3A).

Next, we examined the levels of TNF-α from the murine tumors by ELISA and found that baicalein treatment promoted the production of TNF-α *in vivo*. Similarly, the increase of TNF-α secretion after baicalein treatment was also observed *in vitro* (Figure 3B, ****p < 0.0001, *p = 0.0106). To further verify whether TNF-α was involved in baicalein-induced M1 polarization, we neutralized TNF-α by TNF-α neutralizing antibody in THP-1-derived M1 macrophages and found that baicalein-induced increase of M1 surface marker CD86 was partially reversed after TNF-α neutralization (Figure 3C, ****p < 0.0001, ***p = 0.0005).

The activation of the NF-κB signaling pathway has been demonstrated to result in the induction of pro-inflammatory cytokines including TNF-α that are involved in the M1 macrophage polarization (Ben-Neriah and Karin, 2011; Hoesel and Schmid, 2013). On this basis, we determined the influence of baicalein on the NF-κB pathway. Western blotting data showed that *p*-IκB and p-P65 were up-regulated by baicalein treatment in M1 macrophages (Figure 3D). A laser-scanning confocal microscope was used to detect the nuclear transfer of NF-κB P65 and we found that baicalein treatment could markedly increase P65 nuclear translocation in M1 macrophages (Figure 3E). To further confirm that NF-κB signaling was involved in baicalein-induced TNF-α production, we knocked down the P65 gene in M1 macrophages using P65 siRNA transfection and found that the TNF-α level was significantly decreased in P65 knocked-down M1 macrophages after baicalein treatment, as compared with negative control (Figure 3F, ****p < 0.0001).

### Baicalein is a Potential Inhibitor of PI3Kγ

**FIGURE 4:**
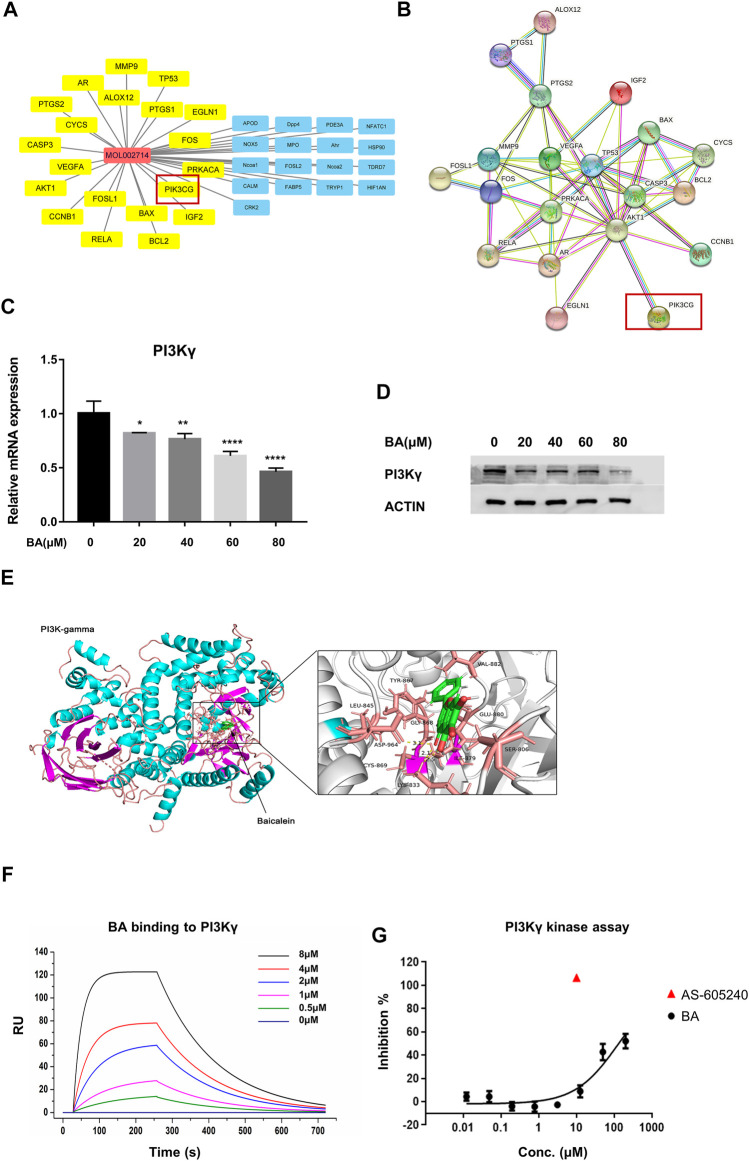
Baicalein is a potential inhibitor of PI3Kγ. **(A)** pharmacological network of baicalein (molecular ID: mol002714) and melanoma targets generated with Cytoscape V3.8.1. The property of yellow nodes are the common targets of drug and disease. All protein targets are represented by their gene symbols. **(B)**, protein-protein interaction (PPI) network of drug and disease target analysis. **(C,D)** the mRNA expression and protein levels of PI3Kγ p110 in THP-1-derived M1 macrophages with or without baicalein treatment. **(E)** the molecular docking of baicalein on PI3Kγ. **(F)** the binding curve of baicalein to PI3Kγ was measured by SPR. **(G)** the inhibitory effect of baicalein on PI3Kγ. Data are presented as mean ± SD and are obtained from at least independent experiments. *p < 0.05, **p < 0.01, ***p < 0.001, ****p < 0.0001, for comparison with control group. BA = baicalein.

To identify the drug-target interaction, the potential targets of baicalein were retrieved from the TCM pharmacology (TCMSP) database and analysis platform (https://old.tcmsp-e.com/tcmsp.php). A total of 37 baicalein targets (Figure 4A) were subsequently mapped to the Pharmacogenomics Knowledge Base (PharmGKB, https://www.pharmgkb.org/), the Therapeutic Target Database (TTD, http://db.idrblab.net/ttd/), the DrugBank (https://go.drugbank.com/), the GeneCards (https://www.genecards.org/) and Online Mendelian Inheritance in Man (OMIM, https://www.omim.org/) to obtain the associated targets in melanoma. There were 22 targets that were associated with melanoma (Figures 4A,B).

Among these targets, PI3KCG (Phosphatidylinositol-4,5-Bisphosphate 3-Kinase Catalytic Subunit Gamma, also known as PI3Kγ) has been demonstrated as one of the key molecules that modulate the immune function of macrophages in tumors. Furthermore, it has been reported that PI3Kγ inhibited the anti-cancer immunity and promoted the immunosuppressive functions of TAMS by attenuating the activation of the NF-κB pathway (De Henau et al., 2016; Kaneda et al., 2016). Our qRT-PCR and western blotting results showed that baicalein treatment markedly suppressed the PI3Kγ in mRNA and protein levels in THP-1 derived macrophages (Figures 4C,D; *p = 0.0244, **p = 0.0046, ****p < 0.0001). We also utilized the docking grogram GOLD Suite v5.3 to obtain information regarding the docking ability of baicalein to PI3Kγ. The optimal binding conformation of the baicalein-PI3Kγ complexes was presented in Figure 4E and the binding energy value of baicalein was −8.873 kcal/mol, which predicted good binding activity to PI3Kγ. The localized SPR analysis further confirmed baicalein’s binding affinity on PI3Kγ (Figure 4F). Next, the *in vitro* protein kinase assay for PI3Kγ demonstrated that baicalein inhibited PI3Kγ in a dose-dependent manner (Figure 4G).

## Discussion

The application of baicalein, as a main bioactive ingredient extracted from the root of medical herb *Scutellaria baicalensis*, has been studied in the treatment of a variety of cancers, such as breast cancer, NSCLC, nasopharyngeal carcinoma, hepatocellular carcinoma, and the underlying mechanisms mainly focus on inducing apoptosis and autophagy, cell cycle arrest, inhibiting proliferation and invasion of tumor cells (Bie et al., 2017; Yan et al., 2018; Guo et al., 2019; Nik Salleh et al., 2020; Zhang et al., 2020). In addition to malignant cells, increasing evidence demonstrated the dominant role of the large proportions of immune cells within the TME in orchestrating tumor progression and resistance to anti-tumor drugs (Cassetta and Pollard, 2018). In response to the stimuli of tumor-derived factors, TAMs abundant in the TME of most cancers can be switched into an immunosuppressive phenotype to inhibit antitumor responses of T cells (Li et al., 2019). Selectively depletion of TAMs and reprogramming immunosuppressive M2-like TAMs to antitumor M1 phenotypes has been explored for anti-tumor therapies (Arlauckas et al., 2017; Cassetta and Pollard, 2018). So far, to our knowledge, the immunomodulatory effects of baicalein on TAMs in the tumor mass remain elusive. In this study, we revealed the anti-tumor activity of baicalein in melanoma and breast cancer, and determined the mechanism through the immune cells in TME.

Our results identified that baicalein significantly increased the percentage of tumor-killing M1 phenotype in the TME of melanoma and breast cancer. To determine if baicalein could directly regulate macrophage polarization, we treated THP-1-derived macrophages with baicalein and found that baicalein not only induced M1 macrophage polarization but also enhanced the production of M1-related factors, such as IRF1, KYNU, CD86, IL-1β, and TNF-α. Regarding that immune profiles of different murine solid tumors are associated with tumorigenesis and prognosis (Yu et al., 2018), we chose mice bearing B16-F10 melanoma and triple-negative breast cancer 4T1 to explore the treatment effects of baicalein, because both tumor models have been recognized as poorly immunogenic (Aznar et al., 2019). Inherently low levels of immune infiltration including macrophages (CD45 + CD11b + F4/80+) were presented in B16-F10 melanoma, and myeloid cells (CD11b+) from 4T1 tumors were found to exert suppress proliferation of T cells more than myeloid cells from B16-F10 (De Henau et al., 2016; Aznar et al., 2019). We found that baicalein significantly inhibited tumor growth in both tumor-bearing mice, and the therapeutic effects were more attributed to TAMs skewing to the M1 phenotype. M1-like macrophages were described as highly phagocytic and highly inflammatory and could facilitate anti-tumor immune response in the TME by secreting nitric oxide, reactive oxygen species (ROS), and inflammatory cytokines, including TNF-α, IL-1 and IL-6 (Brown et al., 2017; Yang et al., 2020).

Although it has been reported that baicalein exhibited an inhibitory effect on M2-TAMs, we did not observe any significant changes in the percentage of M2 phenotype *in vivo*, and baicalein treatment did not alter the M2-related markers *in vitro* as well (data not shown). The different results of baicalein on M2-like phenotype in these two studies might be due to the utilization of different tumor mice models. Zhao et al. (2018) used nude mice bearing MDA-MB-231 human breast cancer cells mixed with THP-1 human monocyte-derived macrophages, while we used mice bearing B16-F10 murine melanoma cells and 4T1 murine breast cancer cells. Zhao et al. (2018) mice were immunodeficient, while the immune system of our mice was not compromised.

Next, we determined the mechanism of baicalein on the M1 phenotype polarization by RNA-seq analysis and found that baicalein could increase TNF-α production in M1 macrophages *via* the NF-κB pathway. TNF-α is one of the major M1-related markers. We also observed that inhibition of TNF-α by TNF-α neutralized antibody partially reversed the baicalein-induced M1 polarization. Under the stimuli of LPS and IFN-γ, the activation of the NF-κB pathway is crucial for the activation of M1-like TAMs (Martinez and Gordon, 2014). Consistent with this, we found that baicalein further increased the phosphorylation levels of IκB and P65 and enhanced the nuclear translocation of P65 in the LPS/IFN-γ induced M1-like macrophages. In addition, the P65 gene knockdown suppressed expression of TNF-α in cells treated with baicalein, which further supported that baicalein switched TAMs to immunostimulatory M1 phenotype via NF-κB/TNF-α signaling.

As innate immune cells and antigen-presenting cells, the activated M1 exerts its tumor-killing function not only by direct cytotoxicity and production of inflammatory cytokines and chemokines, but also by increasing the recruitment of T cells and enhanced tumor-specific T helper 1 (Th1) response (Martinez and Gordon, 2014; House et al., 2020). It has been reported that CXCL9/CXCL10 production derived from macrophages can orchestrate T cell recruitment and activation (House et al., 2020), and we observed significantly elevated levels of CXCL9/CXCL10 in M1 phenotype cells treated with baicalein, however, the percentage of T cells in TME was not influenced by baicalein. Given that the results of RNA-seq indicated an upregulation of hypoxia-inducible factor 1 (HIF-1) pathway in baicalein-treatment cells (Figure 3A) and HIF-1 has been recognized as an important negative modulator of T cell response (McNamee et al., 2013), therefore, we suspected that such metabolic parameter might lead to the dysfunctional tumor-infiltrating T cell states. Furthermore, our results showed that baicalein treatment did not alter the percentage of CD206 + M2-TAMs, and recent studies revealed that the long-lasting interaction between CD206 + macrophages and CD8+ T cells impeded the T cell migration into the tumor site (Peranzoni et al., 2018), which may also elucidate the no alternation on T cell infiltration.

PI3Kγ has been reported as a key molecule in macrophage polarization, and inhibition of PI3Kγ activates the NF-κB-related inflammatory signals (De Henau et al., 2016; Kaneda et al., 2016). Therefore, we assessed whether PI3Kγ was a potential target of baicalein. Our pharmacological network analysis predicted that PI3Kγ might be one of the major targets of baicalein in the tumor and the results from the docking program and LSPR confirmed that baicalein displayed good binding activity to PI3Kγ. We further found that baicalein not only exhibited a direct inhibitory effect on the protein kinase activity of PI3Kγ, but also reduced the mRNA and protein expression of PI3Kγ, indicating that baicalein might be a novel PI3Kγ inhibitor. It has been reported that PI3Kγ inhibitor, such as IPI-549, could promote the anti-tumor effect of anti-PD-1 antibody by remodeling myeloid cell infiltration, especially TAMs in tumors, which provides a rationale to consider that baicalein, as a potential PI3Kγ inhibitor, might overcome the drug resistance to immune checkpoint blocking (ICB) antibodies (De Henau et al., 2016; Kaneda et al., 2016). Further studies are needed to verify this hypothesis.

**FIGURE 5:**
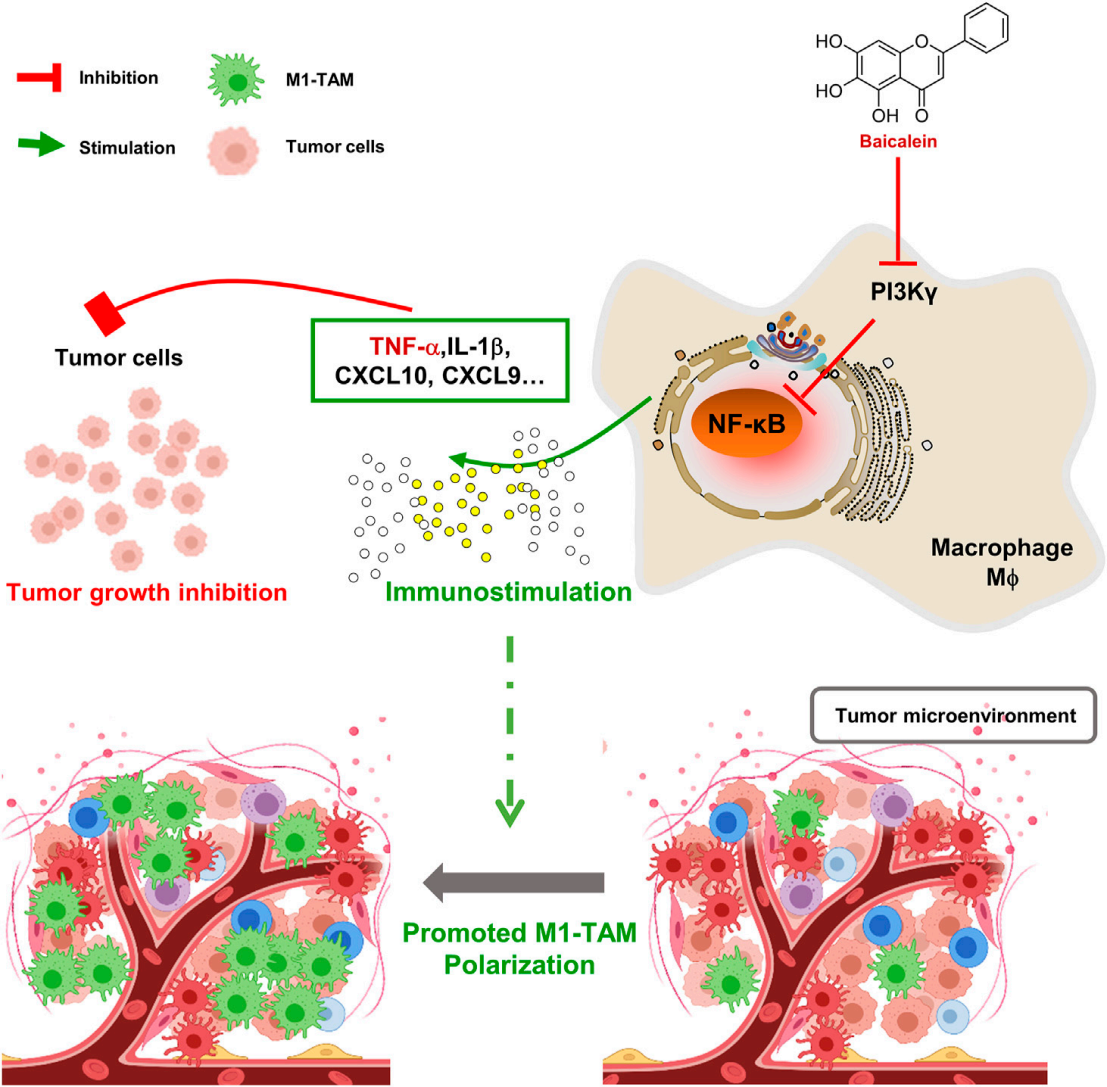
Schematic diagram of mechanism about baicalein in promoting macrophage toward M1 polarization. BA = baicalein.

In summary, baicalein mediated the TAMs skewing to M1-TAMs in TME, and then retarded tumor growth. These effects, at least in part, were linked to the PI3Kγ/NF-κB signaling pathway(Figure 5). Therefore, this study provides new insights into the mechanism of the baicalein-mediated antitumor effect.

## Data Availability

The datasets presented in this study can be found in online repositories. The names of the repository/repositories and accession number(s) can be found below: Sequence Read archive (SRA), PRJNA750043.

## Supplementary Material

Supplementary Figure S1 Flow cytometry discrimination for TAMs by cell surface and intracellular markers. A gating strategy showing the detailed sequence of flow plots. Immune cells were gated as follows: **(A,B)** single cells were gated by FSC-A, SSC-A, and FSC-H out of the events from **(A)**; **(C)** dead cells stained with FVS700 were excluded; **(D)** CD45+ cells were gated from the live cells; **(E)** from CD45+ cells, CD11b+ monocytes were further assessed; **(F)** M1-TAMs were separated based on the expression of CD11c and F4/80 from **(E)**; **(G)**, M2-TAMs were designed as CD206+ F4/80+ cell population from **(E)**.

Supplementary Figure S2 **(A–G)** Flow cytometry gating strategy for T cells in tumor tissues. Immune cells were gated as follows: live, CD45+CD3+CD4+T cells (F); live, CD45+CD3+CD8+T cells **(F)**; live, CD45+CD3+CD4+Foxp3+CD25+ Treg cells **(G)**. Quantification results of CD4+T cells, CD8+ T cells, and Treg cells in tumor tissues from B16-F10 and 4T1 tumor-bearing mice after baicalein treatment were shown in **(H–L)**. Data are presented as mean ± SD. NS, no significance. BA=baicalein.
