# Supplementary material for: Baicalein Potentiated M1 Macrophage Polarization in Cancer Through Targeting PI3Kγ/ NF-κB Signaling
Source: Front Pharmacol. 2021 Aug 25;12:743837. doi: 10.3389/fphar.2021.743837 (PMC8423900; doi:10.3389/fphar.2021.743837)
Supplement: Supplementary file 1 [file Table1.DOCX]

Supplementary Table 1.

| Gene | Forward (5’ to 3’) | Reverse (5’ to 3’) |
| --- | --- | --- |
| Human actin | AAGGTGACAGCAGTCGGTT | TGTGTGGACTTGGGAGAGG |
| Human PI3Kγ | CACCCAAAAGCATATCCTAAGC | GTAATGCAGAACATCATCGTCC |
| Human P65 | CCAGACCAACAACAACCCCTTCC | AAGCAGAGCCGCACAGCATTC |
| Human KYNU | GCAGTTGGAAATGTTGAACTCT | CCCACTAATGCAGGTTTAATCG |
| Human CXCL9 | AAGACCTTAAACAATTTGCCCC | TGCTGAATCTGGGTTTAGACAT |
| Human CXCL10 | CTCTCTCTAGAACTGTACGCTG | ATTCAGACATCTCTTCTCACCC |
| Human IRF-1 | CGGGGCTCATCTGGATTAATAA | GGTCTTTCACCTCCTCGATATC |
| Human TNF-α | CAATGGCGTGGAGCTGAGAG | TCTGGTAGGAGACGGCGATG |

Primer for qRT-PCR
