# Supplementary figures and images for: Baicalein Potentiated M1 Macrophage Polarization in Cancer Through Targeting PI3Kγ/ NF-κB Signaling
Source: Front Pharmacol. 2021 Aug 25;12:743837. doi: 10.3389/fphar.2021.743837 (PMC8423900; doi:10.3389/fphar.2021.743837)

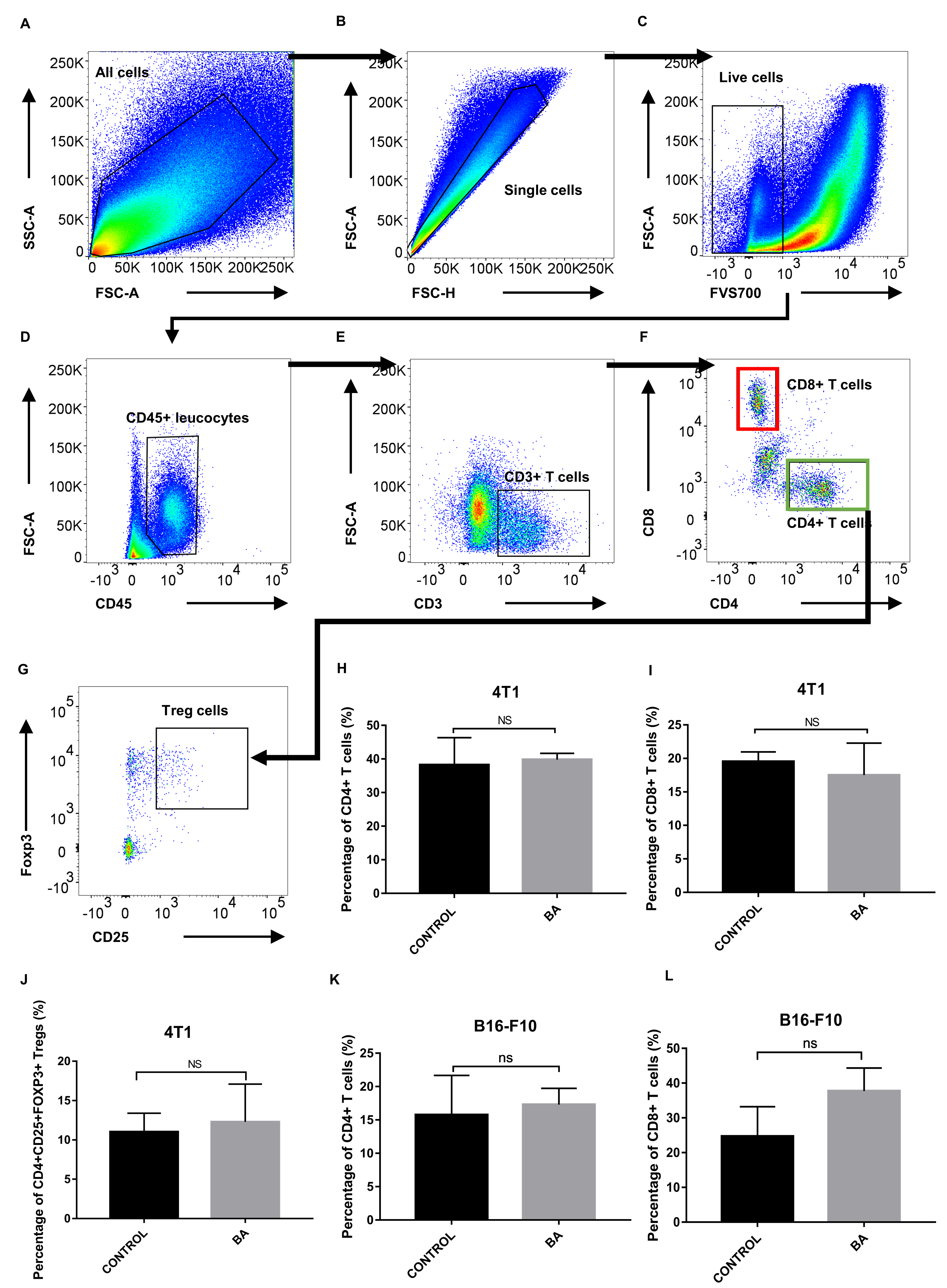

Supplement: Supplementary file 2 [file Image2.TIF]

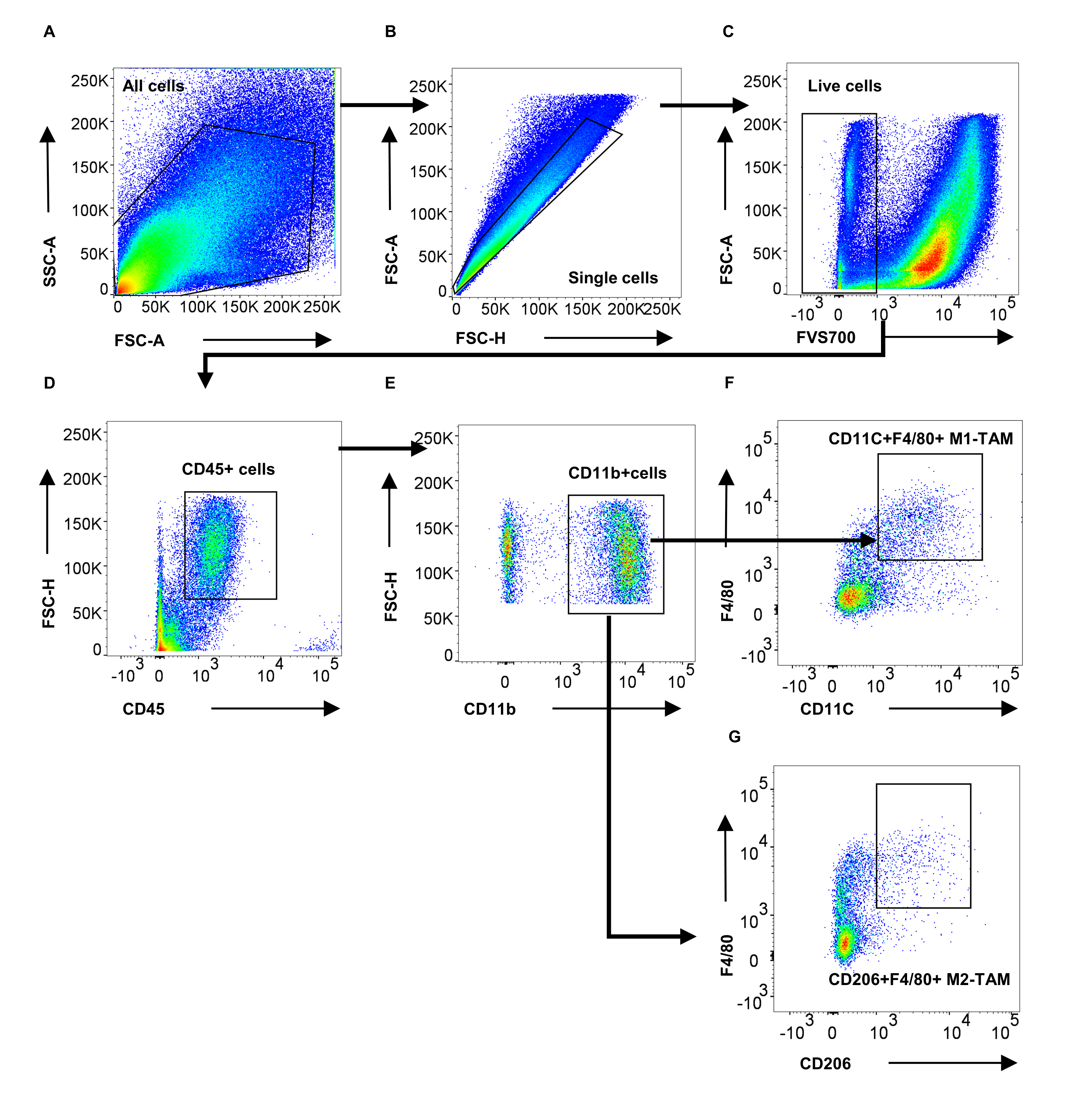

Supplement: Supplementary file 3 [file Image1.TIF]
