# Supplementary material for: Baicalein Potentiated M1 Macrophage Polarization in Cancer Through Targeting PI3Kγ/ NF-κB Signaling
Source: Front Pharmacol. 2021 Aug 25;12:743837. doi: 10.3389/fphar.2021.743837 (PMC8423900; doi:10.3389/fphar.2021.743837)
Supplement: Supplementary file 4 [file DataSheet1.DOCX]

Supplementary information files:

The datasets presented in this study can be found in online repositories. We have uploaded the RNA-seq data (Fig.3) onto the accessible resource the Sequence Read Archive (SRA), and the accession number is PRJNA750043. For direct access to data, please click this web link: <https://www.ncbi.nlm.nih.gov/bioproject/?term=PRJNA750043> .

For source data, we have uploaded the data to <https://www.jianguoyun.com/>, the download links are attached below:

1. The source data for Figure.1:

<https://www.jianguoyun.com/p/Db6xZbAQheDYCRiLpYQE>

1. The source data for Figure.2:

<https://www.jianguoyun.com/p/Df_OSbcQheDYCRiMpYQE>

1. The source data for Figure.3:

<https://www.jianguoyun.com/p/Da48g8cQheDYCRiNpYQE>

1. The source data for Figure.4:

<https://www.jianguoyun.com/p/DXK03_IQheDYCRiOpYQE>

1. The source data for supplementary figure 2:

<https://www.jianguoyun.com/p/Dc1Kj4MQheDYCRiPpYQE>
